# Associations between dietary folate intake and risks of esophageal, gastric and pancreatic cancers: an overall and dose-response meta-analysis

**DOI:** 10.18632/oncotarget.18775

**Published:** 2017-06-28

**Authors:** Wen Liu, Heng Zhou, Yaoqi Zhu, Chaorong Tie

**Affiliations:** ^1^ Department of Pathology, Renmin Hospital of Wuhan University, Wuhan, 430060, Hubei Province, P. R. China; ^2^ Department of Stomatology, Zhongnan Hospital of Wuhan University, Wuhan, 430071, Hubei Province, P. R. China; ^3^ Department of Stomatology, Taikang Tongji Hospital, Wuhan, 430000, Hubei Province, P. R. China

**Keywords:** esophageal cancer, gastric cancer, pancreatic cancer, dose-response analysis, meta-analysis

## Abstract

There are still some controversies on the association between dietary folate intake and the risk of upper gastrointestinal cancers including esophageal, gastric and pancreatic cancers. Hence, a comprehensive meta-analysis on all available literatures was performed to clarify the relationship between dietary folate intake and risks of upper gastrointestinal cancers. An electric search was performed up to December 12^th^, 2016 within the PubMed, MEDLINE AND EMBASE databases. Ultimately, a total of 46 studies which evaluated the association between folate intake and risks of upper gastrointestinal cancers were included. According to the data from included studies, the pooled results showed significant association between folate intake and esophageal (OR = 0.545, 95%CI = 0.432-0.658), gastric (OR=0.762, 95%CI=0.648-0.876) and pancreatic (OR=0.731, 95%CI=0.555-0.907) cancers. Linearity dose-response analysis indicated that with 100μg/day increment in dietary folate intake, the risk of esophageal, gastric and pancreatic cancers would decrease by 9%, 1.5% and 6%, respectively. These findings indicated that higher level of dietary folate intake could help for preventing upper gastrointestinal cancers including esophageal, gastric and pancreatic cancers.

## INTRODUCTION

Folate, also named vitamin B9, is a naturally occurring nutrient and is found in many foods including fruits, vegetables legumes, cereals, and liver. Human can’t produce folate *de novo* and need to uptake folate from dietary intake. Evidences implicated deficient folate is related to increased risks of many cancers [1].

Folate plays an important role in the process of DNA synthesis, repair, and methylation, and was hypothesized to decrease risks of gastrointestinal cancers. The main carcinogenesis mechanisms of folate are inducing DNA strand breaks by causing uracil mis-incorporation into DNA and changing levels of DNA methylation [2]. These aberrant changes may result in potential alterations of critical proto-oncogene and tumor suppressor gene expressions [3]. Animal experiments referring mice and dogs suggested that high levels of folate intake affected DNA methylation and eventually decreased the risks of gastric cancer [4, 5]. In addition, the polymorphisms of genes in folate metabolizing pathway may modulate the susceptibility of several cancers.

Previous studies have summarized published data and indicated that increased folate intake was associated with the increased risks of prostate [6] and breast [7] cancers, but decreased the risks of colorectal [8] and cervical [9] cancers. Two previous meta-analysis have estimated the associations of folate intakes and risks of esophageal, gastric and pancreatic carcinomas and indicated that increased folate intakes were associated with decreased risks of esophageal and pancreatic cancers [10, 11]. However, the results of these studies about the relationship between folate intake and gastric cancer risk remained inconsistent. Larsson et al. indicated that increased folate intake were associated with decreased risks of cardia and non-cardia gastric cancers [11]. Basing on more studies, another systematic review showed no relationship between dietary folate intake and risks of gastric cancers [10]. Therefore, to clarify the associations between folate intake and upper gastrointestinal cancers and evaluate the dose-response relationship between them, we performed an overall meta-analysis based on current observational studies.

## RESULTS

### Summary of studies’ characteristics

**Figure 1:**
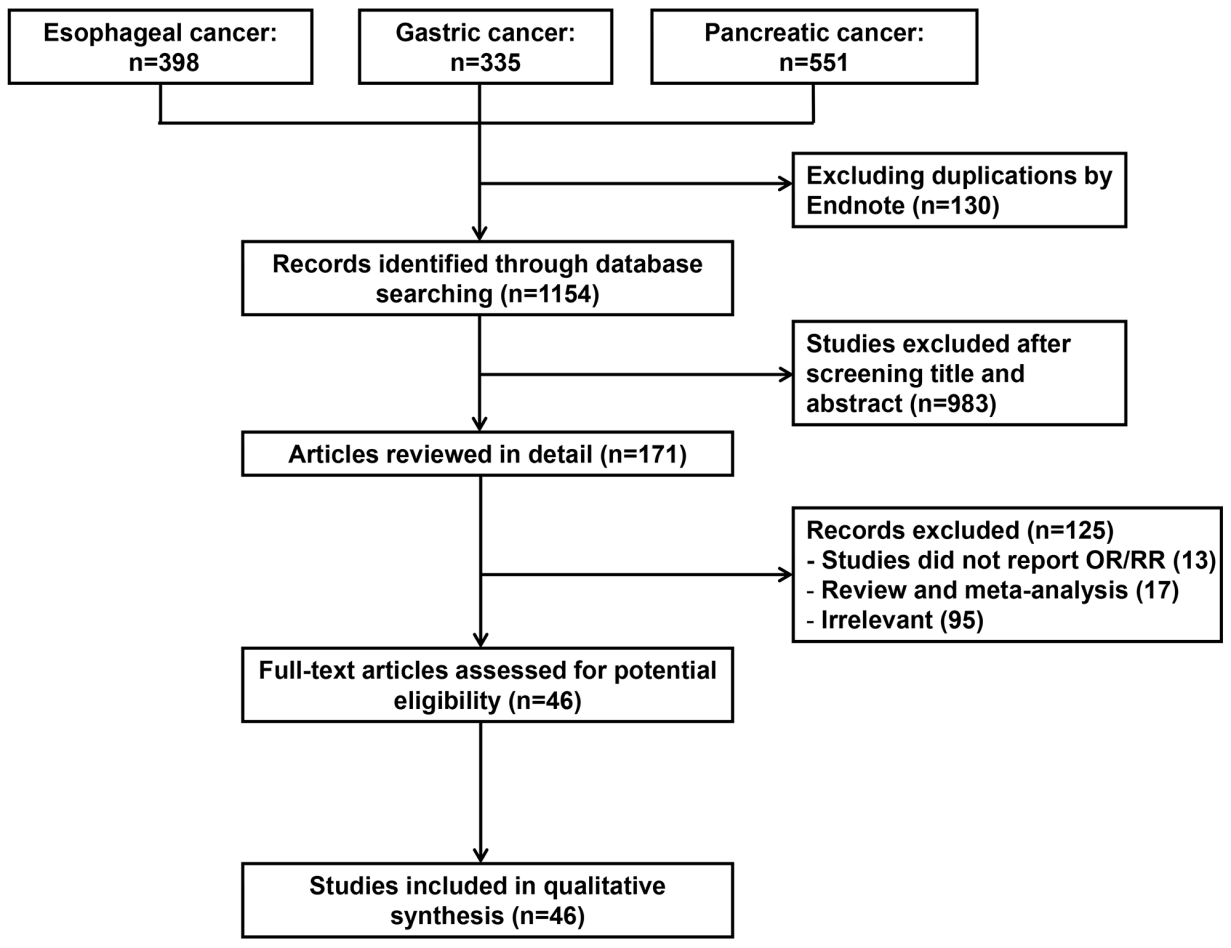
Flow chart of the literature search used in this meta-analysis

Total 1284 studies were collected from our initial search including studies about esophageal cancer (n=398), gastric cancer (n=335) and pancreatic cancer (n=551). After duplicates automatically removing with EndNote, total 1154 potential articles were remained. Then, after screening titles and abstracts, 983 irrelevant studies were excluded; the remained 171 records, which investigated the associations between upper gastrointestinal cancers and folate intake, were eligibly evaluated with full text reading. Based on our inclusive criteria mentioned in Materials and Methods, 46 articles were eventually included in our meta-analysis. Among all the selected studies, 19 were conducted in patients of esophageal cancer [12-30], 21 were in patients of gastric cancer [12, 14, 15, 22, 26, 28, 31-45] and 12 were in patients of pancreatic cancer [46-57]. Figure 1 shows the eligible selecting process. Main characteristics of all include articles were showed in Table 1.

**Table 1:** Characteristics of studies included in the meta-analysis

Studies	Country	Study Design	Year	Age	Sex	Sample Size (cases/ controls)	Disease type	Exposure range (μg/day)	Measurement	Dose-response
2014 Xiao	USA	Cohort	1995-2004	50-71	M/W	GC: 939/492292 EC: 759/492292	GC/EC	566 vs 288	FFQ (Supplement and diet)	No
2014 Chen	China	Case-control	2008-2011	-	M/W	767/765	GCA/Non-GCA	>310 vs <230	FFQ (Diet)	Yes
2013 Gao	China	Case-control	2008-2012	28-76	M/W	264/535	Non-GCA	>310 vs < 230	FFQ (Diet)	Yes
2011 Aune	Uruguay	Case-control	1996-2004	23-89	M/W	GC: 275/2032 EC: 234/2032	GC	275.31 vs 123.83	FFQ (Diet)	Yes
2010 Epplein	China	Cohort	1996-2006	40-70	M/W	338/136442	GCA/Non-GCA	>346.5 vs <218.7	FFQ (Diet)	Yes
2009 Pelucchi	Italy	Case-control	1997-2007	22-80	M/W	230/547	GC	The highest vs the lowest quintile	FFQ (Diet)	No
2005 Kim	Korea	Case-control	1997-1998	-	M/W	136/136	GCA/Non-GCA	>354 vs <234	FFQ (Diet)	No
2003 Nomura	USA	Case-control	1993–1999	-	M/W	300/446	GC	>315 vs <236	FFQ (Diet)	No
2002 Chen	USA	Case-control	1988-1994	-	M/W	GC: 154/449 EC: 124/449	GC/EC	The highest vs the lowest quintile	FFQ (Diet)	No
2000 Botterweck	Netherlands	Cohort	1986-1992	55-69	M/W	310/120852	GC	>384.16 vs <201.96	FFQ (Diet)	Yes
2006 Larsson	Sweden	Cohort	1987-2004	40-76	W	156/61433	GC	>260 vs < 203	FFQ (Supplement and diet)	No
2001 Mayne	USA	Case-control	1993-1995	30-79	M/W	GC: 607/687 EC: 488/687	GC/EC	The highest vs the lowest quintile	FFQ (Diet)	No
2001 Munoz	Venezuela	Case-control	1991-1997	>35	M/W	302/485	GC	The highest vs the lowest quintile	FFQ (Diet)	No
1999 Lizbeth	Mexico	Case-control	1989-1990	24-88	M/W	220/752	GC	>466.26 vs <257.4	FFQ (Diet)	Yes
1994 Vecchia	Italy	Case-control	1985-1992	19-74	M/W	723/2024	GC	>262 vs <163	FFQ (Diet)	Yes
1997 Harrison	USA	Case-control	1992-1994	-	M/W	31/132	GC	The highest vs the lowest quintile	FFQ (Diet)	No
2004 Lissowska	Poland	Case-control	1994-1996	-	M/W	274/463	GC	The highest vs the lowest quintile	FFQ (Diet)	No
2016 Ren	China	Cohort	1985-1991	40-69	M/W	GC: 255/29584 ESCC: 498/29584	GC/ESCC	The highest vs the lowest quintile	serum	No
2015 Chang	China	Case-control	2000	>20	M/W	GC: 206/415 EC: 218/415	GC/EC	The highest vs the lowest quintile	serum	No
2007 Vollset	Europe	Case-control	1992-1998	42.7-71.4	M/W	245/631	GCA/Non-GCA	The highest vs the lowest quintile	serum	No
2014 Lee	China	Case-control	1998-2006	-	M/W	149/155	GC	The highest vs the lowest quintile	serum	No
2015 Fanidi	Europe	Case-control	1992-2000	41-71	M/W	ESCC: 126/255 EAC: 26/274	ESCC/EAC	The highest vs the lowest quintile	serum	No
2013 Sharp	Northern Ireland	Case-control	2002-2005	<85	M/W	223/256	EAC	≥421 vs ≤318	FFQ (Supplement and diet)	No
2013 Huang	China	Case-control	2010-2012	-	M/W	126/167	ESCC	The highest vsthe lowest quintile	serum	No
2012 Tavani	Italy	Case-control	1991-2009	-	M/W	505/22828	EC	≥312.5 vs ≤257.3	FFQ (Diet)	Yes
2011 Zhao	China	Case-control	2008-2010	-	M/W	155/310	ESCC	>300 vs <230	FFQ (Diet)	Yes
2011 Jessri	Iran	Case-control	-	40-75	M/W	47/96	ESCC	The highest vsthe lowest quintile	FFQ (Diet)	No
2011 I biebele	Australia	Case-control	2003-2006	18-79	M/W	267/393	ESCC/EAC	379 vs 196	FFQ (Diet)	Yes
2006 Galeone	Italy	Case-control	1992-1999	<80	Men	351/875	ESCC	The highest vs the lowest quintile	FFQ (Diet)	No
2006 De Stefani	Uruguay	Case-control	1996-2004	40-89	M/W	234/1032	ESCC	The highest vs the lowest quintile	FFQ (Diet)	No
2005 Yang	Japan	Case-control	2001-2004	18-80	M/W	165/495	EC	>400 vs <300	FFQ (Diet)	Yes
2002 Bollschweiler	Germany	Case-control	1997-2000	-	M/W	117/100	ESCC/EAC	>164 vs <100	EBIS (Diet)	Yes
2013 Bao	China	Case-control	2010-2011	-	M/W	106/106	ESCC	The highest vs the lowest quintile	serum	No
1988 Brown	USA	Case-control	1982-1984	<79	M	74/157	EC	The highest vs the lowest quintile	FFQ (Supplement and diet)	No
2011 Chuang	Europe	Cohort	1994	25-70	M/W	638/520000	PC	The highest vs the lowest quintile	serum	No
2011 Bravi	Italy	Case-control	1991-2008	34-80	M/W	326/652	PC	The highest vs the lowest quintile	FFQ (Diet)	No
2010 Oaks	USA	Cohort	1993-2001	55-74	M/W	266/51988	PC	The highest vs the lowest quintile	FFQ (Supplement and diet)	No
2009 Keszei	Netherlands	Cohort	1986-1999	55-69	M/W	363/120852	PC	>259.1 vs <176.3	FFQ (Diet)	Yes
2009 Gong	USA	Case-control	1995-1999	21-85	M/W	532/1701	PC	≥738 vs <280	FFQ (Supplement and diet)	No
2007 Schernhammer	USA	Case-control	1989-1990	40-75	M/W	247/740	PC	The highest vs the lowest quintile	serum	No
2006 Larsson	Sweden	Cohort	1987-1990	45-83	W	135/81922	PC	≥350 vs <200	FFQ (Diet)	Yes
2004 Skinner	USA	Cohort	1976-1986	40-75	M/W	187/125480	PC	≥500 vs <300	FFQ (Supplement and diet)	Yes
2001 Stolzenberg	Finland	Cohort	1985-1988	50-69	M/W	157/27101	PC	≥373 vs <280	FFQ (Diet)	Yes
1999 Stolzenberg	Finland	Case-control	1985-1988	50-69	M/W	126/247	PC	The highest vs the lowest quintile	serum	No
2016 Huang	China	Cohort	1993-1998	45-74	M/W	271/63257	PC	207 vs 108	FFQ (Diet)	Yes
2009 Anerson	Canada	Case-control	2003-2007	<75	M/W	422/312	PC	Folate supplement vs non-folate supplement	FFQ (Supplement and diet)	No

Abbreviations: EBIS, ErnahrungsBeratungs und Informations-System; EC, Esophageal Cancer; EAC, esophageal adenocarcinoma; ESCC, Esophageal squamous cell cancer; FFQ, food frequency questionnaire; GC, Gastric Cancer; GCA, Gastric cardiac adenocarcinoma; PC, Pancreatic Cancer.

### Esophageal cancer

Probands of 4 studies were in American participants [15, 26, 28, 29], 5 in Chinese [12, 14, 17, 19, 30] and 5 in Europeans [13, 16, 18, 23, 27]. In terms of the study design, 2 were cohort studies [12, 15, 18] and 17 were case-control studies [13, 14, 16-25, 58]. Seven studies clearly reported patients with Esophageal squamous cell cancer (ESCC) [12, 13, 15, 17, 19-21, 23, 24, 27, 28, 30] and six studies were about esophageal adenocarcinoma (EAC) [13, 15, 16, 21, 26-28]. Eleven studies investigated dietary folate intake from food [18-28] and 3 studies further examined dietary folate intake from food and supplement [15, 16, 29]. Five studies reported detecting folate concentration in serum samples from patients [12-14, 17, 30]. Six case-control studies [18, 19, 21, 22, 25, 27] and 1 cohort study [15] which evaluated the association between dietary folate intake without supplement and risk of esophageal cancer were included in dose-response analysis. Two studies didn’t set the lowest dose concentration group as reference group [15, 27]. The reference group transformation has been described above.

**Figure 2:**
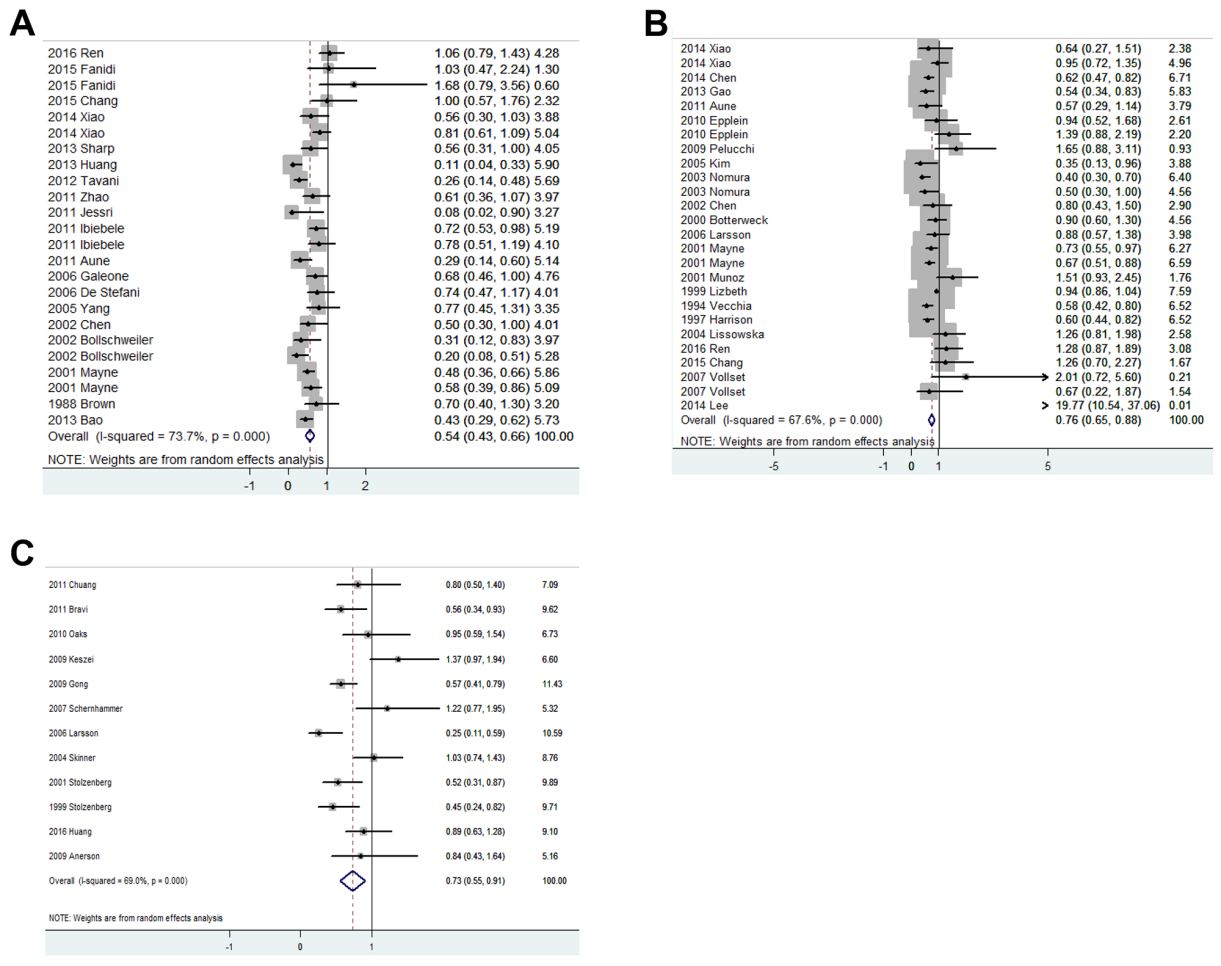
Forest plots of the association between dietary folate intake and risk of esophageal cancer (A), gastric cancer (B) and pancreatic cancer (C)

To assess the relationship between the risk of esophageal cancer and dietary folate intake, total 19 studies including 2036 patients and 7086 controls were collected. The forest plot is shown in Figure 2A. Significant heterogeneity (p<0.001, I^2^ = 73.7%) between these studies suggested that a random effect model was selected. The pooled results showed that dietary folate intake comparing highest levels vs. lowest levels was associated with the decreased risk of esophageal cancer (odds ratio (OR) = 0.545, 95% confidence interval (CI) = 0.432-0.658, Table 2).

**Table 2:** Results including overall and subgroup analysis of pooled OR, 95%CI, heterogeneity test and publication bias

Overall and subgroup analysis	Numbers of studies	Pooled OR	95%CI	Heterogeneity Test			Publication Bias (*P*)	
				*Q*	*P*	I^2^, %	Egger’s test	Begg’s test
**Esophageal cancer**								
Total	20	0.545	0.432-0.658	87.57	<0.001	73.7	0.027	0.023
**Study design**								
Cohort	2	0.821	0.569-1.073	4.11	0.128	51.4	0.466	0.602
Case-control	17	0.496	0.386-0.606	59.90	<0.001	68.3	0.080	0.130
**Histological type**								
ESCC	7	0.551	0.370-0.731	51.39	<0.001	80.5	0.152	0.091
EAC	6	0.561	0.373-0.749	20.15	0.003	70.2	0.141	0.142
**Country**								
USA	4	0.573	0.474-0.673	5.70	0.336	12.3	0.573	0.708
China	5	0.596	0.255-0.938	36.06	<0.001	91.7	0.174	0.125
Europe	5	0.443	0.238-0.647	15.91	0.014	62.3	0.348	0.125
Others	6	0.770	0.450-1.310	15.35	0.009	67.4	0.188	0.043
**Measurement**								
Diet	11	0.547	0.426-0.667	33.92	0.001	61.7	0.01	0.01
Supplement and diet	3	0.692	0.530-0.853	1.99	0.574	0	0.412	0.327
Serum	5	0.708	0.329-1.088	40.56	<0.001	87.7	0.458	0.117
**Gastric cancer**								
Total	21	0.762	0.648-0.876	77.08	<0.001	67.6	0.808	0.270
**Study design**								
Cohort	5	0.967	0.801-1.134	4.46	0.615	0	0.548	0.652
Case-control	16	0.696	0.563-0.829	65.83	<0.001	72.7	0.960	0.248
**Histological type**								
GCA	3	0.729	0.531-0.927	1.14	0.566	0	0.590	0.117
Non-GCA	4	0.681	0.549-0.813	4.09	0.252	26.6	0.761	1
Other GC	17	0.796	0.646-0.947	70.20	<0.001	74.4	0.725	0.278
**Country**								
USA	5	0.627	0.539-0.715	11.11	0.134	37.0	0.510	0.621
Europe	5	0.889	0.562-1.215	9.70	0.084	48.5	0.226	0.573
China	7	0.864	0.579-1.149	22.58	0.002	69.0	0.236	0.322
Others	4	0.859	0.552-1.166	9.76	0.021	69.3	0.885	1
**Measurement**								
Diet	18	0.714	0.591-0.836	60.25	<0.001	71.8	0.216	0.622
Supplement and diet	2	0.884	0.654-1.115	0.76	0.683	0	0.015	0.043
Serum	4	1.217	0.475-1.960	9.65	0.047	58.6	0.849	0.624
**Sex**								
Women	3	0.857	0.405-1.309	6.01	0.050	66.7	0.416	0.602
Men	2	0.599	0.088-1.109	2.98	0.085	66.4	0.656	0.251
**Pancreatic cancer**								
Total	12	0.731	0.555-0.907	35.44	<0.001	69.0	0.089	0.054
**Study design**								
Cohort	7	0.800	0.512-1.089	28.43	<0.001	78.9	0.029	0.015
Case-control	5	0.589	0.456-0.722	6.01	0.198	33.5	0.829	1
**Country**								
USA	4	0.885	0.565-1.206	9.08	0.028	67.0	0.604	0.497
Europe	5	0.457	0.326-0.588	5.75	0.218	30.5	0.069	0.050
Others	3	1.006	0.759-1.252	2.94	0.230	32.0	0.709	0.602
**Measurement**								
Diet	8	0.669	0.450-0.888	21.93	0.001	72.6	0.156	0.099
Supplement and diet	5	0.756	0.559-0.952	6.65	0.156	39.8	0.831	0.49
Serum	3	0.763	0.338-1.189	5.84	0.054	65.7	0.068	0.117
**Sex**								
Men	5	0.856	0.709-1.003	1.97	0.742	0	0.836	1
Women	5	0.716	0.557-0.874	2.89	0.577	0	0.563	0.624

Abbreviations: EC: Esophageal Cancer; EAC: esophageal adenocarcinoma; ESCC: Esophageal squamous cell cancer; GC: Gastric Cancer; GCA: Gastric cardiac adenocarcinoma; OR: odds ration; CI: confidence interval.

Table 2 showed the results of specific subgroup analysis based on study designs, countries, histological type and folate intake measurement. All these results were similar in subgroup analysis suggested that folate intake were comprehensive associated with reduced risk of esophageal cancer.

**Figure 3:**
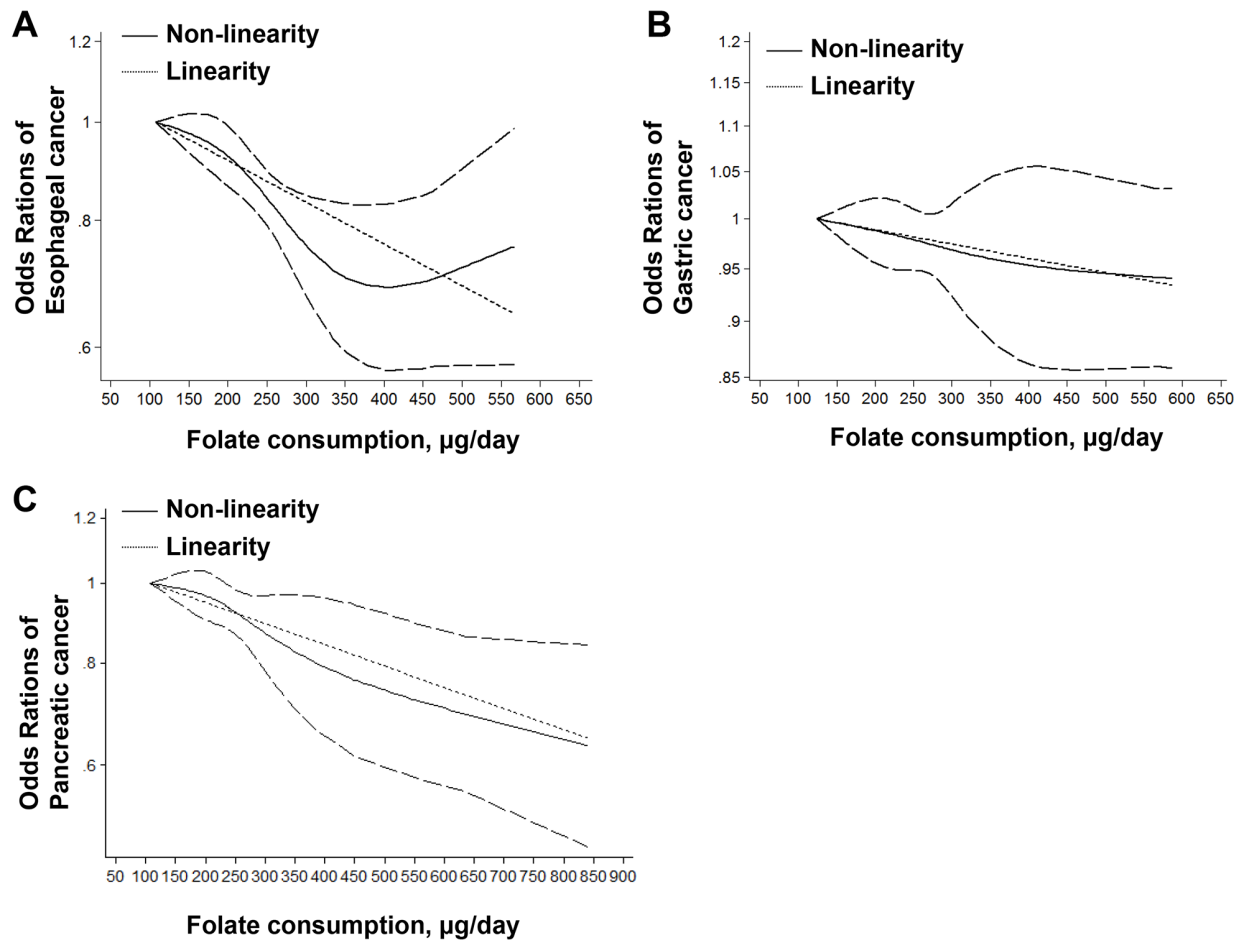
Linearity and non-linearity relationships between dietary folate intake and risk of esophageal cancer (A), gastric cancer (B) and pancreatic cancer (C)

As shown in Figure 3A, the linearity test of dose-response analysis suggested that with increased 100 μg/day folate intake from diet, the risk of esophageal cancer decreased 9% degree (OR=0.91, 95%CI=0.88-0.94). The non-linearity test (*p*<0.001) indicated that the lowest risk of esophageal cancer was at the dose of 405 μg/day (OR=0.69, 95%CI=0.57-0.83). After the dose of folate intake > 405 μg/day, the risk of esophageal cancer would increase after the fall.

### Gastric cancer

Totally 5 studies were about American participants [15, 26, 28, 37, 42, 43], 5 were about European participants [35, 39, 42, 44, 58] and 7 were about Chinese participants [12, 14, 22, 31, 32, 34, 45]. In terms of the study design, 5 were cohort studies [12, 15, 34, 38, 39] and 16 were case-control studies [14, 22, 26, 28, 31, 32, 35-37, 40-45, 58]. Three studies clearly reported patients with gastric cardiac adenocarcinoma (GAC) [15, 28, 44] and 4 studies were about Non-GAC [15, 28, 32, 44]. Eighteen studies investigated dietary folate intake from food [22, 26, 28, 31, 32, 34-38, 40-43, 58] and 2 studies further examined dietary folate intake from food and supplement [15, 39]. Four studies reported detecting folate concentration in serum samples from patients [12, 14, 44, 45]. Two studies have respectively investigated the association between folate intake and risk of gastric cancer by sex [34, 37]. One study only included women participant [39]. Five case-control studies [22, 31, 32, 41, 42] and four cohort studies [15, 34, 38, 39] which evaluated the associations between dietary folate intake and risks of gastric cancer were included in dose-response analysis. One study didn’t set the lowest dose concentration group as reference group [15]. The reference group transformation has been described above.

As shown in Figure 2B, 5 cohort studies and 16 case-control studies were collected to analyze the association between dietary folate intake and risk of gastric cancer. The comprehensive pooled relative risk (RR) indicated a significant association between increased folate intake and decreased risk of gastric cancer (OR=0.762, 95%CI=0.648-0.876, Table 2). There was a significant heterogeneity (*p*<0.001, I^2^=67.6%) which suggested a further subgroup analysis.

Table 2 showed the results of specific subgroup analysis based on study designs, countries, histological type, folate intake measurement and sex. When stratified by cohort studies, 5 studies were included and indicated no statistically significant association existing between dietary folate intake and risk of gastric cancer (OR=0.967, 95%CI=0.801-1.134). The pooled OR of case-control studies suggested a high dietary folate intake was associated with a statistically significant decreased risk of gastric cancer (OR=0.696, 95%CI=0.563-0.829). Subgroup analysis by country demonstrated that there was a significant association between increased folate intake with decreased risk of gastric cancer in Americans (OR=0.627, 95%CI=0.539-0.715) and no associations in Chinese (OR=0.864, 95%CI=0.579-1.149), Europeans (OR=0.889, 95%CI=0.562-1.215) and other countries (OR=0.859, 95%CI=0.552-1.166). Subgroup analysis by histological type indicated that increased dietary folate intake were significantly associated both with Gastric cardiac adenocarcinoma (GCA) (OR=0.729, 95%CI=0.531-0.927) and non-GCA (OR=0.681, 95%CI=0.549-0.813). Subgroup analysis by measurement suggested that high dietary folate intake from diet was associated with a statistically significant decreased risk of gastric cancer (OR=0.714, 95%CI=0.591-0.836). However, there was no association between high dietary folate intake from diet and supplement and risk of gastric cancer (OR=0.884, 95%CI=0.654-1.115). Detecting folate levels in serum suggested that there was no association between folate intake and risk of gastric cancer (OR=1.217, 95%CI=0.475-1.960). Increased folate intake was associated with decreased risk of gastric cancer in men (OR=0.599, 95%CI=0.088-1.109, but not in women (OR=0.857, 95%CI=0.405-1.309).

As shown in Figure 3B, non-linearity (p=0.20) dose-response analysis indicated no relationship between folate intake from diet and risk of gastric cancer. However, a linearity relationship (p=0.03) was found and suggested that 1.5% decrease of gastric cancer for each 100 μg/day increase of dietary folate intake (OR=0.985, 95%CI=0.972-0.998).

### Pancreatic cancer

Probands of 4 studies were in American participants [48, 50, 51, 53], 5 in Europeans [46, 47, 52, 54, 55] and 5 in other countries. In terms of the study design, 7 were cohort studies [46, 48, 49, 52-54, 56] and 5 were case-control studies [47, 50, 51, 55, 57]. Eight studies investigated dietary folate intake from food [47-50, 52, 56] and 5 studies further examined dietary folate intake from food and supplement [48, 50, 53, 54, 57]. Three studies reported detecting folate concentration in serum samples from patients [46, 51, 55]. Five studies have respectively investigated the association between folate intake and risk of pancreatic cancer by sex [46-48, 53, 56]. Total 7 studies were included in dose-response analysis [48-50, 52-54, 56].

As shown in Figure 2C, 7 cohort studies and 5 case-control studies were collected to analyze the association between dietary folate intake and risk of pancreatic cancer. The comprehensive pooled RR indicated a significant association between increased folate intake and decreased risk of pancreatic cancer (OR=0.731, 95%CI=0.555-0.907, Table 2). There was a significant heterogeneity (p<0.001, I^2^=69.0%) which suggested a further subgroup analysis.

Table 2 showed the results of specific subgroup analysis based on study designs, countries, folate intake measurement and sex. The pooled result of cohort studies suggested a weak association existing between dietary folate intake comparing highest levels vs. lowest levels and decreased risk of pancreatic cancer (OR = 0.800, 95%CI = 0.512-1.089). The pooled OR of case-control studies suggested a high dietary folate intake was associated with a statistically significant decreased risk of pancreatic cancer (OR=0.589, 95%CI=0.456-0.722). Subgroup analysis by country demonstrated that there was a significant association between increased folate intake with decreased risk of pancreatic cancer in Europeans (OR=0.457, 95%CI=0.326-0.588) and no associations in Americans (OR=0.885, 95%CI=0.565-1.206) and other countries (OR=1.006, 95%CI=0.759-1.252). Evaluating the association between risks of pancreatic cancer and increased folate intake from diet with (OR=0.756, 95%CI=0.559-0.952) or without supplement (OR=0.669, 95%CI=0.450-0.888) suggested that a superfluous folate supplement is not needed. Detecting folate levels in serum suggested that there was a statistically significant association between folate intake and risk of pancreatic cancer (OR=0.763, 95%CI=0.338-1.189). Increased folate intake was associated with decreased risk of pancreatic cancer in women (OR=0.716, 95%CI=0.557-0.874), but not in men (OR=0.856, 95%CI=0.709-1.003).

As shown in Figure 3C, the linearity test of dose-response analysis suggested that with increased 100 μg/day folate intake from diet, the risk of pancreatic cancer decreased 6% degree (OR=0.94, 95%CI=0.92-0.97,). The non-linearity test (*p*<0.001) also indicated that the risk of pancreatic cancer decreased with folate intake increasing.

### Sensitivity analysis and publication bias

One included study of this meta-analysis was omitted each time to evaluate the stability of pooled results. The results remained similar when any result was removed from the pooled results in this meta-analysis. Begg’s test and Egger’s test were used to evaluate the publication bias, the results were summarized in Table 2. There were significant publication biases in the results which evaluate the associations between folate intake and esophageal cancer (Egger’s test: p=0.027; Begg’s test: p=0.023); esophageal cancer in diet (Egger’s test: p=0.01; Begg’s test: p=0.01); pancreatic cancer in cohort subgroup analysis (Egger’s test: p=0.029; Begg’s test: p=0.015) and gastric cancer in supplement and diet subgroup analysis (Egger’s test: p=0.015; Begg’s test: p=0.043). The trim-and-fill method was used to re-calculate the publication bias. All the new results remained similar to the original results. These results were considered as steady.

## DISCUSSION

Folate is a water-soluble B vitamin and is found in many foods including fruits, vegetables legumes, cereals, and liver. Human can’t produce folate *de novo* and need to uptake folate from dietary intake [1, 59]. Folate plays an important role in the process of DNA synthesis, repair, and methylation, and was hypothesized to decrease risks of gastrointestinal cancers. Two main mechanisms of folate deficiency leads to carcinogenesis: (1) by leading complete convention of dUMP to dTMP, which makes mis-incorporation of uracil into DNA and induces breaks and mutations of chromosome; and/or (2) inducing alternations in expression of critical proto-oncogenes and tumor suppressor genes by causing aberrant methylated level of DNA [2, 3]. In addition, the polymorphisms of 5,10-methylenetetrahydrofolate reductase, a critical junction protein in folate metabolizing pathway by leading folate metabolites to DNA methylation pathway and away from the DNA synthesis pathway, can regulate the susceptibilities of several cancers [60-62].

Our meta-analysis found that increased folate intake was associated with reduced risks of upper gastrointestinal cancers including esophageal, gastric and pancreatic cancers. The dose-response further certified their relationship. Subgroup analysis indicated that the comprehensive inverse associations between dietary folate intake and esophageal cancer. Our data suggested different relationships between dietary folate intake and cancer risks in country, study design, disease type, measurement and sex subgroup analysis of gastric and pancreatic cancers.

The results of this meta-analysis showed that increased dietary folate intake significant decreased risk of esophageal cancer. These results are similar to previous study [10, 11, 63]. In the subgroup analysis based on country, histological type, study design and dietary measurement, our results suggested an inverse association between dietary folate intake and risks of esophageal cancer in all subgroups. Interesting, we observed a higher OR which suggested a weaker link between folate intake and esophageal cancer in supplement and diet subgroup than in diet subgroup. These results suggested an extra folate supplement is not needed in diet for preventing esophageal cancer. The results of dose-response analysis also indicated that with the folate intake > 450 μg/day, the risk of esophageal cancer would increase weakly comparing with the lowest OR, which suggested that a redundant and supplementary folate is not necessary. Zhang et al. found that the risk ration of breast cancer decreased when the dose of folate was low. However, with the folate dose increasing, a positive association was found between folate intake and breast cancer risk [7].

Different from previous studies [10, 11], our results showed a significant association between increased dietary folate intake and reduced gastric cancer risk. Although non-linearity model of dose-response analysis suggested no statistically significant association between folate intake and risk of gastric cancer, linearity model indicated a different result (p=0.03) which certificated our comprehensive pooled OR. Meta-analysis of genetic polymorphisms demonstrated that folate deficiency was associated with increased risk of gastric cancer [11, 64, 65]. Folate supplement can reverse methylation deficiency, stop global hypomethylation and prevent gastric carcinogenesis in hypergastrinemic transgenic mice [5]. Subgroup analysis indicated an inverse association between dietary folate intake and gastric cancer risk in case-control studies, but no association in cohort studies. A possible reason is that only 5 cohort studies were included in this analysis. Small number of studies and effects of multiple factors may affect recall bias and selection bias and restrict the precision of the last results. Similar to previous studies [11], our data showed a significant inverse association between folate intake and GCA or non-GCA, and a weak inverse link between folate intake and other gastric cancer. These results suggested that dietary folate intake plays different roles in different gastric cancers. In the subgroup analysis based on country, we observed an inverse association between folate intake and gastric cancer only in USA, but not in other countries. In addition, in the subgroup analysis based on measurement, our results showed an inverse association between folate intake coming from diet and risk of gastric cancer. However, no association between folate intake coming from diet and supplement and risk of gastric cancer was found. These results also suggested that an extra folate supplement is not needed in diet for preventing gastric cancer. And the excessive intake of folate may be a risk for gastric cancer since the highest values of 95%CI > 1.00. Different from previous estimate, serum evaluating suggested an increased risk of gastric cancer with high serum concentration. One possible explanation is that since the number of included studies about serum detection of folate and gastric cancer risk is too small, which provide insufficient statistical power to evaluate the risk. Animal experiments suggested a dual role of folate in cancer carcinogenesis: prevention or promotion, depending on the stage of cell transformation at the time of intervention and the dose of folate supplement [66, 67]. Significant decreased risks of gastric cancers were observed both in men and women with folate intake increased.

Results of previous meta-analysis about folate intake and pancreatic cancer risk were inconsistent. Bao et al. found folate intake was not associated with overall risk of pancreatic cancer using only prospective cohort studies [68]. However, other studies considered increased folate intake was associated with decreased pancreatic cancer risk [11, 69]. Our comprehensive meta-analysis found an inverse association between dietary folate intake and pancreatic cancer risk. Dose-response analysis indicated that a 100 μg/day increment in dietary folate intake was associated with a 6% risk decreasing for pancreatic cancer. Results of subgroup analysis based on country showed an inverse association between folate intake and pancreatic cancer risk in European. However, this association was not found in American and other countries. These results suggested that geographic variation or dietary habit may play an important role in the association. Subgroup analysis by sex indicated that women had higher pancreatic risk with low folate intake when compared with men. Similar to esophageal and gastric cancers, our data showed that an extra folate supplement is not needed in diet for preventing pancreatic cancer.

There are several limitations to current meta-analysis. First, the included studies about esophageal, gastric and pancreatic cancer have few cohort studies which may make influence on the actual result. Since, dose-response analysis didn’t separate cohort and case-control studies. Second, subgroup analysis based on measurement only included diet, diet and supplement and serum. Total folate intake and other folate intake measurements were not evaluated for lack of related studies. Third, significant heterogeneity were detected between the studies included in quantitative synthesis. Through further subgroup analysis, we still can’t find all the origin of heterogeneity. Forth, this meta-analysis used pooled results for lacking of individual data, which prevents us from finishing a more precise analysis. Last, some subgroup analysis which included small number of studies may not represent objective and exact results. Hence, our results should be treated as exploratory and with caution.

In conclusion, results of current meta-analysis indicated that higher level of dietary folate intake could help for preventing upper gastrointestinal cancers including esophageal, gastric and pancreatic cancers. Dose-response analysis indicated that with 100μg/day increment in dietary folate intake, the risk of esophageal, gastric and pancreatic cancers would decrease by 9%, 1.5% and 6%, respectively. In addition, our analysis indicated that more well-designed studies about associations between esophageal, gastric and pancreatic cancers and folate intake are necessary for further accurately evaluating subgroup analysis based on country, measurement, histological type and sex.

## MATERIALS AND METHODS

### Literature search

A systematically search was performed up to May 2^th^, 2017 by two reviewers (H. Z. and Y. Z.) within Pubmed, MEDLINE AND EMBASE, using the terms “folate, folic acid or vitamin B9”, “esophageal, oesophagus, gastric, stomach, or pancreatic” and “cancer, neoplasm or carcinoma”. In addition, we reviewed the reference lists from original reports and manually selected for other available publications. No language restrictions were imposed in the searching process.

### Study selection

The studies were included with the following inclusion criteria: (i) the experimental design was a case-control or cohort study; (ii) studies reported the associations of esophageal, gastric, or pancreatic cancer risk with dietary folate intake from diet, dietary folate intake from diet and supplement and serum levels of folate; (iii) RR, hazard ration (HR) or OR with 95% CI was reported to estimate the relative risk of the highest folate intake vs. lowest folate intake; (iv) patient with disease was identified by histological diagnosis; (v) for dose-response analysis, the number of cases and participants and eligible dose concentration must be provided. The selected studies were only limited in using dietary folate intake as only measurement standard. The most recent study was included for duplicate publications.

### Data extraction

The following information was selected independently by two authors (H. Z. and Y. Z.) according to the criteria listed previously: the first author’s name, publication year, country, study design, total sample size, sex, number of cases, number of controls, lowest folate level, highest folate level, difference between highest and lowest folate levels, measurement, range of exposure, histological type (ESCC, EAC, gastric cardiac adenocarcinoma (GAC); non-GAC), risk estimates and 95%CI for evaluating the highest folate levels vs. lowest folate levels. Adjusted rations were chosen in preference to the rations with the highest number of adjusted variables. For the studies which the reference groups were not the lowest dose concentration, the EXCEL macro document (RRest9) was used for the reference group transforming and data re-calculating according to the instructions [70]. All controversial questions were resolved by asking a third author.

### Statistical analysis

The association of folate intake with esophageal, gastric and pancreatic cancers were examined by the pooled risk estimates (RR or OR) with 95%CI. The heterogeneity test was detected with I^2^ statistic. Cut-off points of I^2^ value for low, moderate and high degrees of heterogeneity were 25%, 50% and 75%, respectively. A fixed effect model was chosen when heterogeneity was negligible, otherwise, the random effects model was chosen [71]. Sensitivity analysis was investigated to assess robust of pooled results by omitting one study each time. The publication bias was determined by the Begg rank correlation test and Egger’s linear regression test [72]. P<0.05 was considered statistically significant, and all p-values were two-sided. The trim-and-fill method was used to re-calculate the publication bias when the P values of Begg test or Egger test >0.05. The new pooled results (RR or OR) were compared with the original results. The results were considered as steady if the new pooled results are similar to the original results. At last, we conducted a dose-response meta-analysis using the correlated natural logs of the RRs or ORs with their standard error (SE) across all folate intake categories [73]. To derive the dose-response curve, restricted cubic splines with four knots at the 5%, 35%, 65% and 95% percentiles of the distribution were used to assess for potential curvilinear relations. All data in this meta-analysis were performed using Stata 12.0 (StataCorp LP, College Station, TX, USA).

## Abbreviations

CI: confidence interval
ESCC: esophageal squamous cell cancer
EAC: esophageal adenocarcinoma
EBIS: ErnahrungsBeratungs und Informations-System
FFQ: food frequency questionnaire
GAC: gastric cardiac adenocarcinoma
HR: hazard ration
OR: odds ratio
PC: Pancreatic Cancer
RR: relative risk

## References

[1] Duthie SJ (2011). Folate and cancer: how DNA damage, repair and methylation impact on colon carcinogenesis. J Inherit Metab Dis.

[2] Blount BC, Mack MM, Wehr CM, MacGregor JT, Hiatt RA, Wang G, Wickramasinghe SN, Everson RB, Ames BN (1997). Folate deficiency causes uracil misincorporation into human DNA and chromosome breakage: implications for cancer and neuronal damage. Proc Natl Acad Sci USA.

[3] Choi SW, Mason JB (2000). Folate and carcinogenesis: an integrated scheme. J Nutr.

[4] Xiao SD, Meng XJ, Shi Y, Hu YB, Zhu SS, Wang CW (2002). Interventional study of high dose folic acid in gastric carcinogenesis in beagles. Gut.

[5] Gonda TA, Kim YI, Salas MC, Gamble MV, Shibata W, Muthupalani S, Sohn KJ, Abrams JA, Fox JG, Wang TC, Tycko B (2012). Folic acid increases global DNA methylation and reduces inflammation to prevent Helicobacter-associated gastric cancer in mice. Gastroenterology.

[6] Tio M, Andrici J, Cox MR, Eslick GD (2014). Folate intake and the risk of prostate cancer: a systematic review and meta-analysis. Prostate Cancer Prostatic Dis.

[7] Zhang YF, Shi WW, Gao HF, Zhou L, Hou AJ, Zhou YH (2014). Folate intake and the risk of breast cancer: a dose-response meta-analysis of prospective studies. PLoS One.

[8] Chuang SC, Rota M, Gunter MJ, Zeleniuch-Jacquotte A, Eussen SJ, Vollset SE, Ueland PM, Norat T, Ziegler RG, Vineis P (2013). Quantifying the dose-response relationship between circulating folate concentrations and colorectal cancer in cohort studies: a meta-analysis based on a flexible meta-regression model. Am J Epidemiol.

[9] Zhou X, Meng Y (2016). Association between serum folate level and cervical cancer: a meta-analysis. Arch Gynecol Obstet.

[10] Tio M, Andrici J, Cox MR, Eslick GD (2014). Folate intake and the risk of upper gastrointestinal cancers: a systematic review and meta-analysis. J Gastroenterol Hepatol.

[11] Larsson SC, Giovannucci E, Wolk A (2006). Folate intake, MTHFR polymorphisms, and risk of esophageal, gastric, and pancreatic cancer: a meta-analysis. Gastroenterology.

[12] Ren J, Murphy G, Fan J, Dawsey SM, Taylor PR, Selhub J, Qiao Y, Abnet CC (2016). Prospective study of serum B vitamins levels and oesophageal and gastric cancers in China. Sci Rep.

[13] Fanidi A, Relton C, Ueland PM, Midttun Ø Vollset SE, Travis RC, Trichopoulou A, Lagiou P, Trichopoulos D, Bueno-de-Mesquita HB, Ros M, Boeing H, Tumino R (2015). A prospective study of one-carbon metabolism biomarkers and cancer of the head and neck and esophagus. Int J Cancer.

[14] Chang SC, Goldstein BY, Mu L, Cai L, You NC, He N, Ding BG, Zhao JK, Yu SZ, Heber D, Zhang ZF, Lu QY (2015). Plasma folate, vitamin B12, and homocysteine and cancers of the esophagus, stomach, and liver in a Chinese population. Nutr Cancer.

[15] Xiao Q, Freedman ND, Ren J, Hollenbeck AR, Abnet CC, Park Y (2014). Intakes of folate, methionine, vitamin B6, and vitamin B12 with risk of esophageal and gastric cancer in a large cohort study. Br J Cancer.

[16] Sharp L, Carsin AE, Cantwell MM, Anderson LA, Murray LJ, and FINBAR Study Group (2013). Intakes of dietary folate and other B vitamins are associated with risks of esophageal adenocarcinoma, Barrett’s esophagus, and reflux esophagitis. J Nutr.

[17] Huang GL, Wang SK, Su M, Wang TT, Cai HZ, Yin H, Sun GJ (2013). Serum folate, MTHFR C677T polymorphism and esophageal squamous cell carcinoma risk. Biomed Environ Sci.

[18] Tavani A, Malerba S, Pelucchi C, Dal Maso L, Zucchetto A, Serraino D, Levi F, Montella M, Franceschi S, Zambon A, La Vecchia C (2012). Dietary folates and cancer risk in a network of case-control studies. Ann Oncol.

[19] Zhao P, Lin F, Li Z, Lin B, Lin J, Luo R (2011). Folate intake, methylenetetrahydrofolate reductase polymorphisms, and risk of esophageal cancer. Asian Pac J Cancer Prev.

[20] Jessri M, Rashidkhani B, Hajizadeh B, Jessri M, Gotay C (2011). Macronutrients, vitamins and minerals intake and risk of esophageal squamous cell carcinoma: a case-control study in Iran. Nutr J.

[21] Ibiebele TI, Hughes MC, Pandeya N, Zhao Z, Montgomery G, Hayward N, Green AC, Whiteman DC, Webb PM, and Study of Digestive Health, and Australian Cancer Study (2011). High intake of folate from food sources is associated with reduced risk of esophageal cancer in an Australian population. J Nutr.

[22] Aune D, Deneo-Pellegrini H, Ronco AL, Boffetta P, Acosta G, Mendilaharsu M, De Stefani E (2011). Dietary folate intake and the risk of 11 types of cancer: a case-control study in Uruguay. Ann Oncol.

[23] Galeone C, Pelucchi C, Levi F, Negri E, Talamini R, Franceschi S, La Vecchia C (2006). Folate intake and squamous-cell carcinoma of the oesophagus in Italian and Swiss men. Ann Oncol.

[24] De Stefani E, Ronco AL, Boffetta P, Deneo-Pellegrini H, Acosta G, Correa P, Mendilaharsu M (2006). Nutrient intake and risk of squamous cell carcinoma of the esophagus: a case-control study in Uruguay. Nutr Cancer.

[25] Yang CX, Matsuo K, Ito H, Shinoda M, Hatooka S, Hirose K, Wakai K, Saito T, Suzuki T, Maeda T, Tajima K (2005). Gene-environment interactions between alcohol drinking and the MTHFR C677T polymorphism impact on esophageal cancer risk: results of a case-control study in Japan. Carcinogenesis.

[26] Chen H, Tucker KL, Graubard BI, Heineman EF, Markin RS, Potischman NA, Russell RM, Weisenburger DD, Ward MH (2002). Nutrient intakes and adenocarcinoma of the esophagus and distal stomach. Nutr Cancer.

[27] Bollschweiler E, Wolfgarten E, Nowroth T, Rosendahl U, Mönig SP, Hölscher AH (2002). Vitamin intake and risk of subtypes of esophageal cancer in Germany. J Cancer Res Clin Oncol.

[28] Mayne ST, Risch HA, Dubrow R, Chow WH, Gammon MD, Vaughan TL, Farrow DC, Schoenberg JB, Stanford JL, Ahsan H, West AB, Rotterdam H, Blot WJ, Fraumeni JF (2001). Nutrient intake and risk of subtypes of esophageal and gastric cancer. Cancer Epidemiol Biomarkers Prev.

[29] Brown LM, Blot WJ, Schuman SH, Smith VM, Ershow AG, Marks RD, Fraumeni JF (1988). Environmental factors and high risk of esophageal cancer among men in coastal South Carolina. J Natl Cancer Inst.

[30] Bao L, Peng J, Huang G, Wang S, Yin H, Wang T, Liu F, Sun G (2013). [The study on the relationship between serum folic acid and vitamin B2 levels and esophageal cancer]. [Article in Chinese]. Wei Sheng Yan Jiu.

[31] Chen J, Yuan L, Duan YQ, Jiang JQ, Zhang R, Huang ZJ, Xiao XR (2014). Impact of methylenetetrahydrofolate reductase polymorphisms and folate intake on the risk of gastric cancer and their association with Helicobacter pylori infection and tumor site. Genet Mol Res.

[32] Gao S, Ding LH, Wang JW, Li CB, Wang ZY (2013). Diet folate, DNA methylation and polymorphisms in methylenetetrahydrofolate reductase in association with the susceptibility to gastric cancer. Asian Pac J Cancer Prev.

[33] Hou L, Wang H, Sartori S, Gawron A, Lissowska J, Bollati V, Tarantini L, Zhang FF, Zatonski W, Chow WH, Baccarelli A (2010). Blood leukocyte DNA hypomethylation and gastric cancer risk in a high-risk Polish population. Int J Cancer.

[34] Epplein M, Shu XO, Xiang YB, Chow WH, Yang G, Li HL, Ji BT, Cai H, Gao YT, Zheng W (2010). Fruit and vegetable consumption and risk of distal gastric cancer in the Shanghai Women’s and Men’s Health studies. Am J Epidemiol.

[35] Pelucchi C, Tramacere I, Bertuccio P, Tavani A, Negri E, La Vecchia C (2009). Dietary intake of selected micronutrients and gastric cancer risk: an Italian case-control study. Ann Oncol.

[36] Kim HJ, Kim MK, Chang WK, Choi HS, Choi BY, Lee SS (2005). Effect of nutrient intake and Helicobacter pylori infection on gastric cancer in Korea: a case-control study. Nutr Cancer.

[37] Nomura AM, Hankin JH, Kolonel LN, Wilkens LR, Goodman MT, Stemmermann GN (2003). Case-control study of diet and other risk factors for gastric cancer in Hawaii (United States). Cancer Causes Control.

[38] Botterweck AA, van den Brandt PA, Goldbohm RA (2000). Vitamins, carotenoids, dietary fiber, and the risk of gastric carcinoma: results from a prospective study after 6.3 years of follow-up. Cancer.

[39] Larsson SC, Giovannucci E, Wolk A (2006). Folate intake and stomach cancer incidence in a prospective cohort of Swedish women. Cancer Epidemiol Biomarkers Prev.

[40] Muñoz N, Plummer M, Vivas J, Moreno V, De Sanjosé S, Lopez G, Oliver W (2001). A case-control study of gastric cancer in Venezuela. Int J Cancer.

[41] López-Carrillo L, López-Cervantes M, Ward MH, Bravo-Alvarado J, Ramírez-Espitia A (1999). Nutrient intake and gastric cancer in Mexico. Int J Cancer.

[42] La Vecchia C, Ferraroni M, D’Avanzo B, Decarli A, Franceschi S (1994). Selected micronutrient intake and the risk of gastric cancer. Cancer Epidemiol Biomarkers Prev.

[43] Harrison LE, Zhang ZF, Karpeh MS, Sun M, Kurtz RC (1997). The role of dietary factors in the intestinal and diffuse histologic subtypes of gastric adenocarcinoma: a case-control study in the U.S. Cancer.

[44] Vollset SE, Igland J, Jenab M, Fredriksen A, Meyer K, Eussen S, Gjessing HK, Ueland PM, Pera G, Sala N, Agudo A, Capella G, Del Giudice G (2007). The association of gastric cancer risk with plasma folate, cobalamin, and methylenetetrahydrofolate reductase polymorphisms in the European Prospective Investigation into Cancer and Nutrition. Cancer Epidemiol Biomarkers Prev.

[45] Lee TY, Chiang EP, Shih YT, Lane HY, Lin JT, Wu CY (2014). Lower serum folate is associated with development and invasiveness of gastric cancer. World J Gastroenterol.

[46] Chuang SC, Stolzenberg-Solomon R, Ueland PM, Vollset SE, Midttun Ø Olsen A, Tjønneland A, Overvad K, Boutron-Ruault MC, Morois S, Clavel-Chapelon F, Teucher B, Kaaks R (2011). A U-shaped relationship between plasma folate and pancreatic cancer risk in the European Prospective Investigation into Cancer and Nutrition. Eur J Cancer.

[47] Bravi F, Polesel J, Bosetti C, Talamini R, Negri E, Dal Maso L, Serraino D, La Vecchia C (2011). Dietary intake of selected micronutrients and the risk of pancreatic cancer: an Italian case-control study. Ann Oncol.

[48] Oaks BM, Dodd KW, Meinhold CL, Jiao L, Church TR, Stolzenberg-Solomon RZ (2010). Folate intake, post-folic acid grain fortification, and pancreatic cancer risk in the Prostate, Lung, Colorectal, and Ovarian Cancer Screening Trial. Am J Clin Nutr.

[49] Keszei AP, Verhage BA, Heinen MM, Goldbohm RA, van den Brandt PA (2009). Dietary folate and folate vitamers and the risk of pancreatic cancer in the Netherlands cohort study. Cancer Epidemiol Biomarkers Prev.

[50] Gong Z, Holly EA, Bracci PM (2009). Intake of folate, vitamins B6, B12 and methionine and risk of pancreatic cancer in a large population-based case-control study. Cancer Causes Control.

[51] Schernhammer E, Wolpin B, Rifai N, Cochrane B, Manson JA, Ma J, Giovannucci E, Thomson C, Stampfer MJ, Fuchs C (2007). Plasma folate, vitamin B6, vitamin B12, and homocysteine and pancreatic cancer risk in four large cohorts. Cancer Res.

[52] Larsson SC, Håkansson N, Giovannucci E, Wolk A (2006). Folate intake and pancreatic cancer incidence: a prospective study of Swedish women and men. J Natl Cancer Inst.

[53] Skinner HG, Michaud DS, Giovannucci EL, Rimm EB, Stampfer MJ, Willett WC, Colditz GA, Fuchs CS (2004). A prospective study of folate intake and the risk of pancreatic cancer in men and women. Am J Epidemiol.

[54] Stolzenberg-Solomon RZ, Pietinen P, Barrett MJ, Taylor PR, Virtamo J, Albanes D (2001). Dietary and other methyl-group availability factors and pancreatic cancer risk in a cohort of male smokers. Am J Epidemiol.

[55] Stolzenberg-Solomon RZ, Albanes D, Nieto FJ, Hartman TJ, Tangrea JA, Rautalahti M, Sehlub J, Virtamo J, Taylor PR (1999). Pancreatic cancer risk and nutrition-related methyl-group availability indicators in male smokers. J Natl Cancer Inst.

[56] Huang JY, Butler LM, Wang R, Jin A, Koh WP, Yuan JM (2016). Dietary intake of one-carbon metabolism-related nutrients and pancreatic cancer risk: the singapore Chinese health study. Cancer Epidemiol Biomarkers Prev.

[57] Anderson LN, Cotterchio M, Gallinger S (2009). Lifestyle, dietary, and medical history factors associated with pancreatic cancer risk in Ontario, Canada. Cancer Causes Control.

[58] Lissowska J, Gail MH, Pee D, Groves FD, Sobin LH, Nasierowska-Guttmejer A, Sygnowska E, Zatonski W, Blot WJ, Chow WH (2004). Diet and stomach cancer risk in Warsaw, Poland. Nutr Cancer.

[59] Persson EC, Schwartz LM, Park Y, Trabert B, Hollenbeck AR, Graubard BI, Freedman ND, McGlynn KA (2013). Alcohol consumption, folate intake, hepatocellular carcinoma, and liver disease mortality. Cancer Epidemiol Biomarkers Prev.

[60] Zhuo X, Ling J, Zhou Y, Zhao H, Song Y, Tan Y (2012). Polymorphisms of MTHFR C677T and A1298C association with oral carcinoma risk: a meta-analysis. Cancer Invest.

[61] He J, Liao XY, Zhu JH, Xue WQ, Shen GP, Huang SY, Chen W, Jia WH (2014). Association of MTHFR C677T and A1298C polymorphisms with non-Hodgkin lymphoma susceptibility: evidence from a meta-analysis. Sci Rep.

[62] Fernández-Peralta AM, Daimiel L, Nejda N, Iglesias D, Medina Arana V, González-Aguilera JJ (2010). Association of polymorphisms MTHFR C677T and A1298C with risk of colorectal cancer, genetic and epigenetic characteristic of tumors, and response to chemotherapy. Int J Colorectal Dis.

[63] Zhao Y, Guo C, Hu H, Zheng L, Ma J, Jiang L, Zhao E, Li H (2017). Folate intake, serum folate levels and esophageal cancer risk: an overall and dose-response meta-analysis. Oncotarget.

[64] Sun L, Sun YH, Wang B, Cao HY, Yu C (2008). Methylenetetrahydrofolate reductase polymorphisms and susceptibility to gastric cancer in Chinese populations: a meta-analysis. Eur J Cancer Prev.

[65] Boccia S, Hung R, Ricciardi G, Gianfagna F, Ebert MP, Fang JY, Gao CM, Götze T, Graziano F, Lacasaña-Navarro M, Lin D, López-Carrillo L, Qiao YL (2008). Meta- and pooled analyses of the methylenetetrahydrofolate reductase C677T and A1298C polymorphisms and gastric cancer risk: a huge-GSEC review. Am J Epidemiol.

[66] Song J, Sohn KJ, Medline A, Ash C, Gallinger S, Kim YI (2000). Chemopreventive effects of dietary folate on intestinal polyps in Apc+/-Msh2-/- mice. Cancer Res.

[67] Song J, Medline A, Mason JB, Gallinger S, Kim YI (2000). Effects of dietary folate on intestinal tumorigenesis in the apcMin mouse. Cancer Res.

[68] Bao Y, Michaud DS, Spiegelman D, Albanes D, Anderson KE, Bernstein L, van den Brandt PA, English DR, Freudenheim JL, Fuchs CS, Giles GG, Giovannucci E, Goldbohm RA (2011). Folate intake and risk of pancreatic cancer: pooled analysis of prospective cohort studies. J Natl Cancer Inst.

[69] Lin HL, An QZ, Wang QZ, Liu CX (2013). Folate intake and pancreatic cancer risk: an overall and dose-response meta-analysis. Public Health.

[70] Hamling J, Lee P, Weitkunat R, Ambühl M (2008). Facilitating meta-analyses by deriving relative effect and precision estimates for alternative comparisons from a set of estimates presented by exposure level or disease category. Stat Med.

[71] Higgins JP, Thompson SG, Deeks JJ, Altman DG (2003). Measuring inconsistency in meta-analyses. BMJ.

[72] Egger M, Davey Smith G, Schneider M, Minder C (1997). Bias in meta-analysis detected by a simple, graphical test. BMJ.

[73] Greenland S, Longnecker MP (1992). Methods for trend estimation from summarized dose-response data, with applications to meta-analysis. Am J Epidemiol.

